# Dynamic self-organisation and pattern formation by magnon-polarons

**DOI:** 10.1038/s41467-023-37919-6

**Published:** 2023-04-18

**Authors:** M. Gidding, T. Janssen, C. S. Davies, A. Kirilyuk

**Affiliations:** 1 FELIX Laboratory, Radboud University, Toernooiveld 7, 6525 ED, Nijmegen, The Netherlands; 2 Radboud University, Institute for Molecules and Materials, Heyendaalseweg 135, 6525 AJ, Nijmegen, The Netherlands

**Keywords:** Magnetic properties and materials, Information storage, Nonlinear phenomena

## Abstract

Magnetic materials play a vital role in energy-efficient data storage technologies, combining very fast switching with long-term retention of information. However, it has been shown that, at very short time scales, magnetisation dynamics become chaotic due to internal instabilities, resulting in incoherent spin-wave excitations that ultimately destroy magnetic ordering. Here, contrary to expectations, we show that such chaos gives rise to a periodic pattern of reversed magnetic domains, with a feature size far smaller than the spatial extent of the excitation. We explain this pattern as a result of phase-synchronisation of magnon-polaron quasiparticles, driven by strong coupling of magnetic and elastic modes. Our results reveal not only the peculiar formation and evolution of magnon-polarons at short time-scales, but also present an alternative mechanism of magnetisation reversal driven by coherent packets of short-wavelength magnetoelastic waves.

## Abstract (web summary)

Increasing the speed of magnetization switching is an obvious pathway to improve spintronic device performance. However, very fast magnetization switching is accompanied by instabilities. Here, Gidding et al study these instabilities using optical pumping, and show that instability generated spin-waves can achieve a high enough amplitude to drive switching of the magnetization, with a distinctive coherent pattern.

## Introduction

Finding methods that enable fast, efficient and long-lasting reversal of magnetisation direction represents a major topic of research in condensed matter physics with obvious technological applications^1^. The most theoretically-straightforward but also practical approach for switching magnetisation involves very-large-amplitude precessional motion^2–4^. However, the dynamics of such reorientation across a large angle are dramatically different from the magnetisation precession in the regime of ferromagnetic resonance (FMR)^5^. Very fast reorientations of magnetisation are inexorably accompanied by instabilities^4,6^ deriving from the spin-wave instability of overexcited FMR (the so-called Suhl instability). It is well understood that the Landau-Lifshitz-Gilbert equation still characterises this motion but the effective dissipation constant considerably exceeds, by order(s) of magnitude, that which is typical of FMR^7^. Such damping is related to the excitation of a large spectrum of spin waves during the precession^5,6,8–10^. The faster the magnetic switching is driven, the more energy is pumped into the system, and therefore the more of it flows into incoherent spin-wave modes with wavenumbers spanning a very broad range^11^.

In addition to the non-linear instabilities arising from the magnetostatic interactions within the magnetic system, an extra channel of non-linearity can be provided by the interaction of the excitation with the lattice^12^. The non-linearity and dispersion of acoustic waves, for example, can be amplified by the presence of small-amplitude magnetisation oscillations at similar frequencies. It is completely unknown, however, how such coupling influences magnetisation switching featuring a large cone angle of precession.

Here, we use multi-scale pump-probe experiments, spanning timescales ranging from nanoseconds to milliseconds, to demonstrate how spin-wave instabilities give rise to distinct pattern formation during the process of magnetic switching. Utilising single infrared pulses derived from a cavity-dumped free-electron laser^13^, we strongly pump optical phonons at resonance, dynamically deforming the structure of the material^14^. This temporarily creates a magnetic anisotropy field that, in turn, drives large-amplitude dynamics and switching of the magnetisation. The large-amplitude precessional motion leads to strong instability of the spin system and generates a sizeable number of spin waves, strongly populating the low-energy part of the magnon spectrum. The subsequent repopulation in the presence of a magneto-elastic interaction leads to effective condensation of the waves into the hybridised magnon-polaron region of the spectrum, observed both experimentally and in micromagnetic simulations^15,16^. Ultimately, these magnon-polaron waves synchronise and achieve such high amplitude that they even induce complete switching of magnetisation at the wave maxima.

## Results and discussion

In our study, we used a magnetic 7.5 μm-thick lutetium- and bismuth-doped yttrium-iron-garnet film, hereafter referred to as Lu:YIG^17^. The Lu and Bi dopants induce strong magneto-optical Faraday rotation and very weak magnetic anisotropy, with the latter giving rise to large spatially-uniform magnetic domains as well as a low FMR frequency. The Lu:YIG sample has a fourfold in-plane anisotropy field of 4 kA m^−1^ which results, in the absence of magnetic fields, in four equilibrium orientations of the magnetisation that are orthogonal to each other in the plane of the film. We perform our experiments at the free-electron laser facility FELIX in the Netherlands^18^. In the pump-probe measurements, we typically pump the sample with single transform-limited micropulses^13^ with a wavelength of 13 μm, an energy of ≈ 80 μJ, and duration of ≈ 2 ps. These pulses were focused to an elliptical spot, with full-width-half-maximum diameter of 300 μm and 130 μm, on the surface of the Lu:YIG sample^19^. The spatial distribution of magnetisation dynamics was magneto-optically probed using defocused pulses of wavelength 532 nm and duration 5 ns, with the latter defining the temporal resolution of the experiment. To increase the magneto-optical contrast between the in-plane magnetisation directions of Lu:YIG, the sample was tilted by ≈ 30^∘^ relative to the probe’s path. The spatial resolution of the obtained magneto-optical images is ≈ 2 μm.

**Fig. 1:**
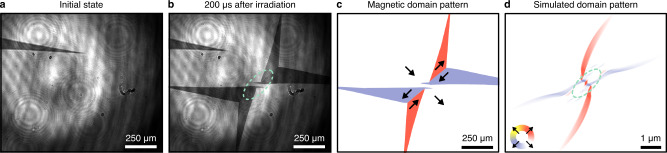
Phononic switching of in-plane magnetisation in Lu:YIG. Magneto-optical images of the magnetisation distribution taken **a** before and **b** 200 μs after irradiation by a single pump pulse of wavelength 13 μm and duration ≈ 2 ps. **c** The switched magnetic domains isolated by subtracting the non-magnetic background. The arrows and associated colour-coding indicate the direction of magnetisation. **d** The calculated impact of a single pump pulse on the magnetisation, obtained via micromagnetic simulations. In **b** and **d**, the dashed green line indicates the half-maximum intensity level of the pump pulse.

After exposing the sample to a single mid-infrared pump pulse, we observe that the homogeneous magnetisation distribution switches to form a peculiar spatial pattern (Fig. 1a, b). The switching consists of four distinct, triangular domains that emanate diagonally outwards from the center of the irradiated region. At the center, however, switching is absent. By subtracting the images taken before and after illumination, we clearly identify that the magnetic domains have two-fold rotational symmetry (Fig. 1c).

The observed four-domain pattern results from large-amplitude magnetisation dynamics^20^ and can be understood in terms of the magnetoelastic interaction. Specifically, the resonant pumping of infrared-active optical phonon modes microscopically displaces the equilibrium atomic positions along an optical phonon coordinate^21^. The effect of such deformation on magnetisation can be modelled using a standard micromagnetic description of magnetoelastic energy^20^. Indeed, simulations of the magnetisation dynamics of Lu:YIG with the inclusion of the magnetoelastic interaction successfully reproduce the switching pattern observed experimentally, as shown in Fig. 1d. The spectral dependence of the switching (shown in the Supplementary Information) confirms the phononic mechanism.

**Fig. 2:**
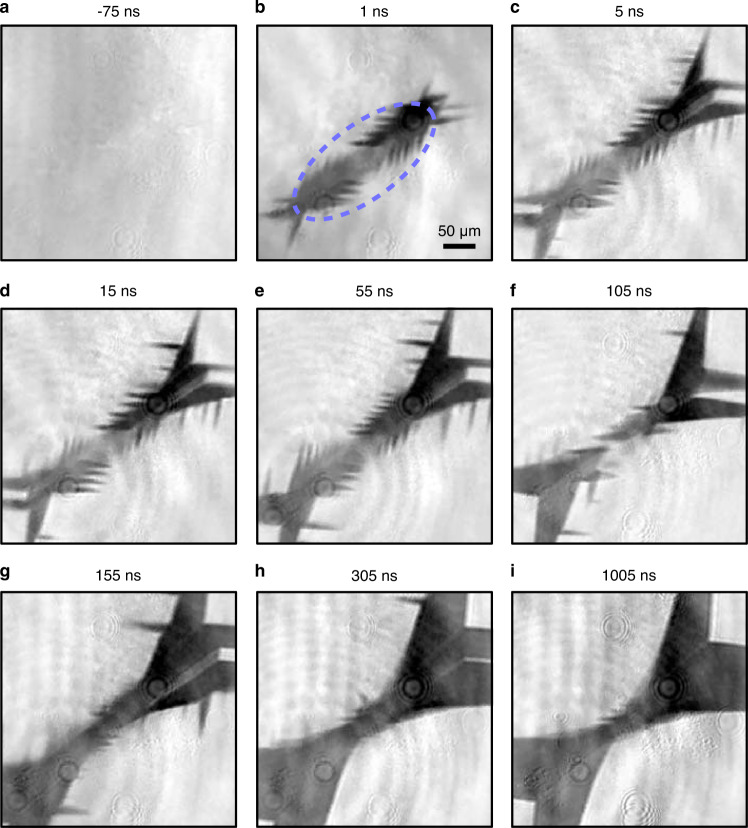
Time-resolved imaging of the pattern formation. **a**–**i** Representative background-subtracted magneto-optical images taken at the indicated times after the arrival of the pump pulse (wavelength 13 μm, duration ≈ 2 ps). The dashed blue line in **b** indicates the half-maximum intensity level of the pump pulse. The circular patterns in the bottom-left corners of **b**–**i** arise from the monochromatic probe pulse scattering off defects in the sample, creating an interference pattern.

To understand how the pattern of magnetic switching forms, we now proceed to single-shot pump-probe measurements, varying the time of arrival of the visible probe with respect to the pump. Typical results of this experiment are shown in Fig. 2. These results show that the triangular domains shown in Fig. 1 emerge and propagate outwards from the center of the irradiated spot, growing in size during the first ≈ 300 ns. More intriguingly, however, during the nascent stages of the pattern formation, we observe a periodic pattern of domains (hereafter referred to as ripples) with a visible period on the order of 10 μm, which is very much unexpected given the large ( ~ 300 μm) diameter of the excitation spot along this axis. During the first hundreds of nanoseconds following the arrival of the pump pulse, Fig. 2 shows that the pattern is remarkably heterogeneous with the ripples propagating outwards from the center. These magnetisation ripples are very reproducible and appear for other orientations of magnetisation in the sample as well (see the Supplementary Information). While the large triangular domains persist for a timescale of hundreds of microseconds to milliseconds (see the Supplementary Information), the ripples have decayed completely within about 300 ns after the arrival of the pump pulse.

It is well understood that a spatially-localised perturbation creates propagating waves with wave vectors determined by the profile of the excitation^16,22^. The results shown in Fig. 2 therefore raise an intriguing question of how an excitation that is reasonably homogeneous (across > 200 μm) results in such a heterogeneous ripple pattern with periodicity on the order of 10 μm. One possible explanation could be provided by the Faraday waves that appear both in fluids and solids that are subject to a periodic vertical oscillation^23,24^. However, taking into account the elastic parameters of YIG crystals together with the excitation frequency, we are able to exclude this possibility.

Instead, we posit that such small features originate from spin-wave instabilities^8–10^ in the presence of magnetoelastic interactions. The magnetisation precession is driven to such a high amplitude that the precessional motion of the magnetisation becomes highly unstable, leading to the generation of a large number of spin waves in a broad frequency spectrum^7,8^. This excitation should therefore lead to chaotic magnetisation dynamics, as shown in Ref. ^6^. However, instead we observe a rather well-defined quasi-periodic pattern of reversed domains, with a periodicity an order of magnitude smaller than what could be expected from the pulse profile. What can be the mechanism creating such a pattern?

**Fig. 3:**
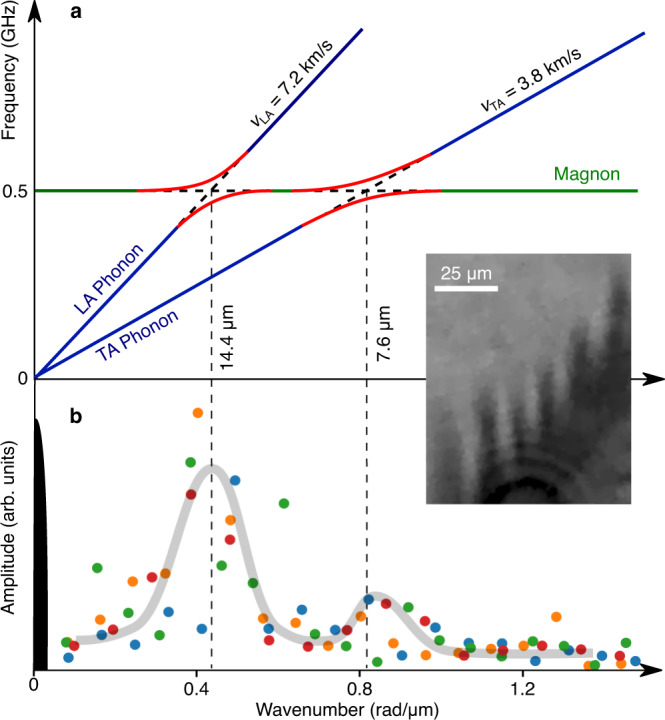
Schematic illustration of the self-accumulation process. **a** Sketch of the anti-crossings (red) between the dispersion relations of the acoustic phonons (blue) and the magnon (green). The group velocities *v* of the longitudinal acoustic (LA) and transverse acoustic (TA) phonon modes are shown. **b** Amplitude of the Fourier transform of the ripple patterns during the first 30 ns (coloured circles) and of the pump pulse (black), with a guide to the eye (grey). The blue, orange, green and red points correspond to the Fourier transforms extracted from the magneto-optical images taken at time delays of 1 ns, 10 ns, 20 ns and 30 ns, respectively. See the Supplementary Information for the data used in the calculation of the Fourier transforms. Inset: representative zoomed section of the magneto-optical image of the ripple pattern used for the Fourier transforms.

Yttrium-iron-garnet films are renowned in the research field of magnonics for their low damping both in the elastic and magnetic subsystems^25,26^. This combination of material properties yield access, via the strong coupling between the two subsystems, to regions in phase space where the magnon and phonon dispersion curves approach and consequently where their frequencies coincide. In these regions, the magnetoelastic coupling combines the magnons and phonons to form magnon-polarons^22^. This quasiparticle hybridisation allows for the efficient transfer of angular momentum, as the slower magnons are dressed with phonons which have a significantly larger group velocity^22,27^. This interaction between the magnetic and elastic subsystems, schematically illustrated in Fig. 3, can be observed directly by, for example, magneto-optics^28,29^.

Brillouin light scattering spectroscopy experiments have previously shown that the thermalisation of an overpopulated magnon gas, in combination with magnon-phonon scattering, leads to the self-organisation of so-called magnetoelastic bosons at the crossing point of the magnon and phonon dispersions^15^. This accumulation phenomenon is confirmed by micromagnetic simulations taking into account the magnetoelastic interaction of magnon-polaron dynamics^16^. Calculations of spin pumping by a parametric excitation also prove that magnons are resonantly enhanced in strength at the magnetoelastic crossing point^30^. Such a collapse of the broad magnon spectrum into the narrow ranges of the magnetoelastic crossing points can explain how, in our case, the broad spectrum resulting from spin-wave instabilities leads to the domain pattern with a well-defined periodicity.

The above supposition is supported by a comparison of the periodicity of magnetic ripples observed during the first 30 ns after excitation (see Fig. 2) with the typical wavenumbers characteristic of magnon-polarons. The ferromagnetic resonance frequency of our sample, given its very weak magnetic anisotropy and small saturation magnetisation, is about 0.5 GHz in the case of magnetic fields close to zero^31^. This, together with the sound velocities of 3.8 km s^−1^ (7.2 km s^−1^) characteristic of transverse (longitudinal) acoustic phonons^32^, leads to an estimated magnon-polaron wavelength of 8 to 15 *μ*m, which corresponds rather well to the smallest observed distances between the ripples. The fact that we observe these waves as reversed domains supports the idea of condensation in the sense that the waves phase-synchronise with each other so that the large amplitude leads to complete magnetisation reversal at the maxima. Moreover, this phenomenon might even resemble neural entrainment, the process through which brain-waves (large-scale electrical oscillations in the brain) naturally synchronise to the rhythm of periodic visual, auditory or tactile stimuli^33–35^.

As an outlook, while our single-shot pump-probe imaging experiments clearly unveil the formation of large-amplitude magnon-polarons at the nanosecond time scale, further experiments must examine more closely the spatiotemporal details of their development at sub-nanosecond timescales. This could in particular provide a better insight into not only the intricacies of phonon-induced switching of magnetisation but also the dynamics of wave-synchronisation in general, which is a phenomenon of broad interest in different areas of science^34,36^.

## Methods

### Materials

The sample studied is a 7.5 μm-thick doped magnetic iron-garnet film^17^ with chemical composition Lu_1.69_Y_0.65_Bi_0.66_Fe_3.85_Ga_1.15_O_12_. The sample was grown on a (001) oriented gadolinium gallium garnet substrate with trace amounts of Pb impurities resulting from the growth process. This sample has a magnetisation^37^ of *M*_*s*_ = 300 kA m^−1^ and a weak in-plane fourfold anisotropy field 4 kA m^−1^ which results, in the absence of magnetic fields, in four equilibrium orientations of the magnetisation that are orthogonal to each other in the plane of the film.

### Magneto-optical single-shot pump-probe microscopy experiments

To excite and detect the magnetisation dynamics of the sample, we use the technique of single-shot pump-probe microscopy, with the pump being delivered by the cavity-dumped laser FELICE^13^ at the free-electron laser facility FELIX in the Netherlands^18^. This laser delivers transform-limited pulses with a central wavelength of 13 μm, duration of ≈ 2 ps and energy ≈ 80 μJ at a rate of 10 Hz. A motorised shutter placed in the path of the infrared laser allows us to select single pulses. Using a 90^∘^ off-axis parabolic mirror, the infrared pump pulse is focused onto the surface of the Lu:YIG sample to an elliptical spot with a full-width-half-maximum diameter of 300 and 130 μm as assessed by the Liu method^19^. The probe pulse is derived from a synchronised frequency-doubled Nd:YAG laser, which delivers linearly-polarised pulses of wavelength 532 nm and duration ≈ 5 ns. Upon transmission through the sample, the probe is gathered by the objective lens, passed through an analyser and detected using a camera. By measuring the rotation of polarisation of the light transmitted through the sample, induced by the magneto-optical Faraday effect, we are able to spatially resolve magnetisation dynamics across the sample. Electronically shifting the time of arrival of the probe pulse provides temporal resolution, limited to 5 ns as given by the probe’s duration^13^. To enhance the magneto-optical contrast between different states of the in-plane magnetisation of Lu:YIG, the sample was tilted at an angle of approximately 30^∘^ relative to the wave vector of the incoming probe pulse.

### Micromagnetic simulations

Micromagnetic simulations were performed using the MuMax^3^ micromagnetic simulation program^38^. The simulated sample dimensions were 10 × 10 × 0.5 *μ*m^3^ with cell size 9.76 × 9.76 × 500 nm^3^, allowing for the inclusion of exchange interactions in the simulation. Literature values for physical constants in Lu:YIG were used, with the exchange constant^39^ set to 3.7 pJ m^−1^ and the saturation magnetisation^37^ to 300 kA m^−1^. A biaxial anisotropy was implemented in MuMax^3^ via the method described by De Clercq et al.^40^ with *K*_b_ = 750 J/m^3^, and easy axes along the (1, 1, 0) and (1, − 1, 0) directions. The Landau-Lifshitz damping constant *α* was set to 0.12, while at the outer 10% of the simulated sample it increased quadratically up to *α* = 10. This was implemented to create an absorbing boundary region^41^.

Strain was introduced into the simulation using the method of Stupakiewicz et al.^20^, which adds a transient modification to the strain tensor of the sample due to the non-linear interaction of phonon modes^14^, mimicking the strain introduced by an infrared laser pulse. The Gaussian standard deviation of the pulse along the (1, 1, 0) and (1, − 1, 0) axes was set to 0.333 μm and 0.766 μm respectively, equivalent to a full-width at half-maximum (FWHM) of 0.784 μm and 1.80 μm respectively. The elliptical aspect ratio is thus set to 2.3, matching that of the pulse used in the experiments. The pulse’s full-duration half-maximum (FDHM), *τ*, is 10 ps, with the peak of the Gaussian pulse striking the sample 50 ps after the simulation commences.

## Supplementary information

Supplementary Information

## Data Availability

The data supporting the findings of this study are available within the article and its supplementary file.
