# Supplementary material for: Dynamic self-organisation and pattern formation by magnon-polarons
Source: Nat Commun. 2023 Apr 18;14:2208. doi: 10.1038/s41467-023-37919-6 (PMC10113182; doi:10.1038/s41467-023-37919-6)
Supplement: Supplementary file 1 — Supplementary Information [file 41467_2023_37919_MOESM1_ESM.pdf]

Dynamic self-organisation and pattern formation by magnon-polarons

SUPPLEMENTARY INFORMATION

M. Gidding[1,2], T. Janssen[1,2], C. S. Davies[1,2*] and A. Kirilyuk[1,2*]

[1]FELIX Laboratory, Radboud University, Toernooiveld 7, 6525 ED Nijmegen, The Netherlands.
[2]Radboud University, Institute of Molecules and Materials, Heyendaalseweg 135, 6525 AJ Nijmegen, The Netherlands.

*Corresponding author(s). E-mail(s): carl.davies@ru.nl; andrei.kirilyuk@ru.nl;

1 Supplementary micromagnetic simulations

1.1 Time-resolved simulations

The simulated temporal evolution of the switched area of magnetisation, presented in Fig. S1, shows that the magnetisation relaxes back to its equilibrium state within 3 ns. The results of the micromagnetic simulations are qualitatively similar to the experimental results shown in the main text, notwithstanding the difference in length and time scales. While the inclusion of magnetoelastic effective magnetic fields reproduce well the overall pattern of magnetic switching, our calculations do not fully incorporate the reciprocal action of elastodynamics on the magnetisation, so our simulations are unable to reproduce the condensation at the magnon-phonon anti-crossings.

The magnetisation switching observed in the simulations decays much faster compared to that observed experimentally. This mismatch in lifetime is explained by the reduced spatial extent of the simulated magnetic film. In the simulations, the sample dimensions were constrained by the requirement to include the exchange interaction, necessitating in-plane cell-sizes smaller than ≈15 nm. Increasing the spatial extent of our simulated sample with such

cell sizes imposes intolerably high computational overheads. This limitation on the simulation dimensions, in turn, reduces the lifetime of the switched domains, due to the stronger "pressure" exerted by domain walls that are closer together [1].

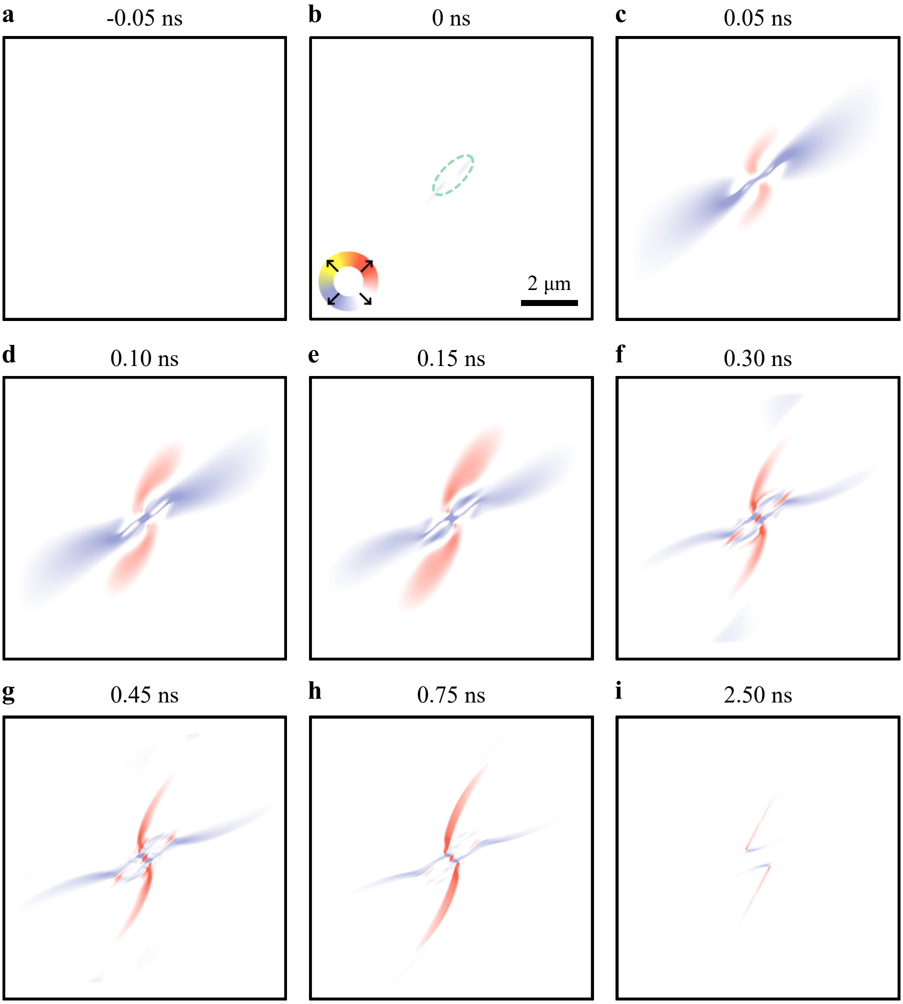

Fig. S1: **(a)-(i)** The direction of the magnetisation in the plane of the simulated sample is shown at several time delays after arrival of the pump pulse as indicated. The colour wheel in panel (b) represents the magnetisation orientation.

2 Supplementary experimental measurements

2.1 Lifetime of the switched magnetisation

The magnetisation switching observed in our experiments was transient in nature. We therefore measured the lifetime of the switched magnetisation, obtaining the results presented in Figs. S2 and S3. The switched magnetisation returns back to its initial orientation within approximately a millisecond after being pumped, with some fluctuation between measurements which is ascribed to the fluctuation in pump pulse intensity. Note the complete absence of ripples on this timescale.

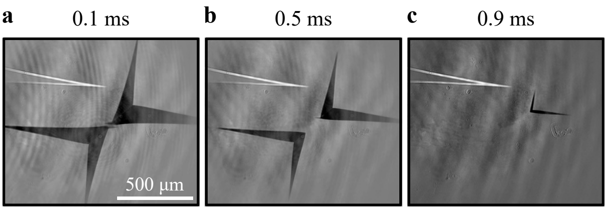

Fig. S2: **(a)-(c)** Magneto-optical images of the magnetisation, taken at different time delays as indicated after the arrival of a pump pulse of wavelength 13.0 µm.

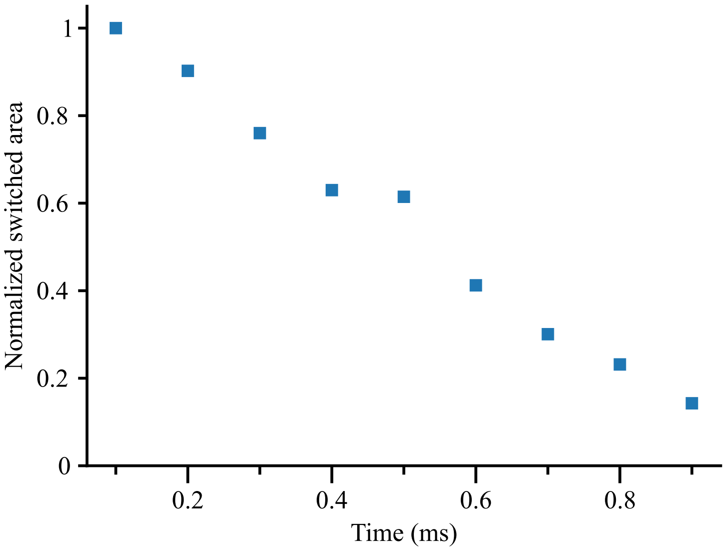

Fig. S3: The normalised size of the switched area of magnetisation as a function of time. Each point represents the average of 20 individual measurements obtained with a pump pulse of wavelength 13 µm.

2.2 Spectral dependence of the switching

The spectral dependence of the area of switched magnetisation, along with that of the LO and TO phonon modes, are shown in Fig. S4. Comparing the two shows the correspondence between magnetic switching and the population of LO phonon modes. The switched area was calculated by taking the ratio of the switched area relative to the total image area, followed by normalisation. The spectral population of the TO and LO phonons are taken from Ref. [2]. We note, however, that these were measured for a differently-doped thin film of YIG, which could explain why the overlap between switching and LO phonon modes is not exact.

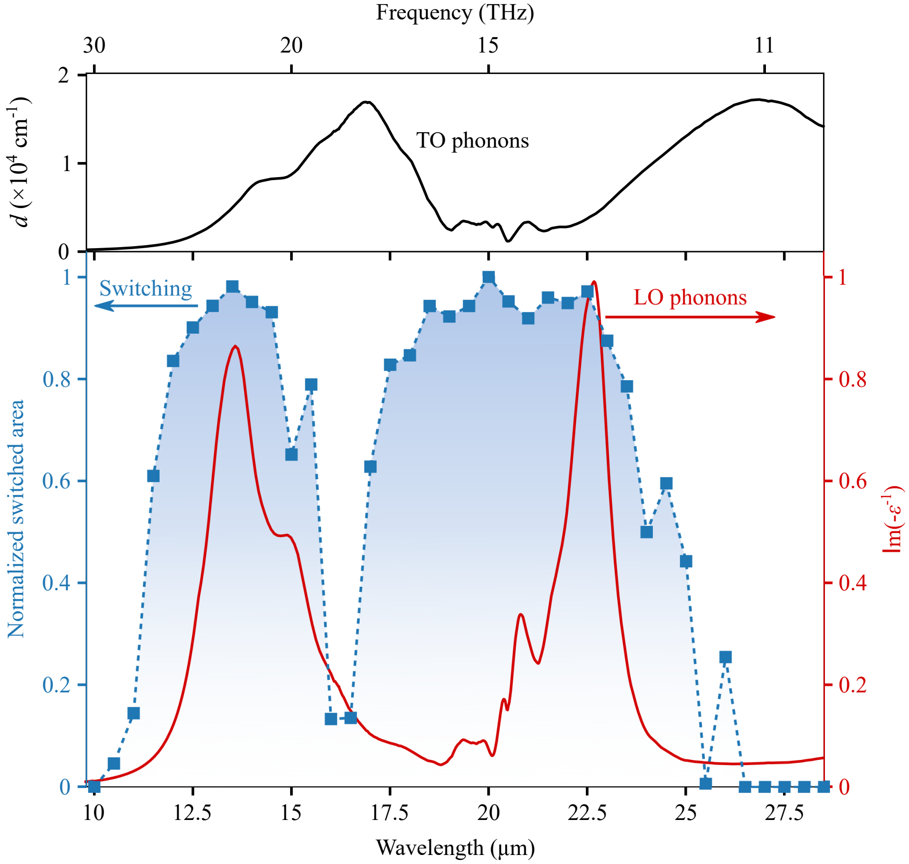

Fig. S4: The normalised size of the switched area of magnetisation as a function of pumping wavelength, where each blue point represents the average of 20 individual measurements. The black and red lines correspond to the spectral population of TO and LO phonons in cobalt-doped YIG [2], defined by the absorption d (top panel) and the loss function $\mathrm{Im}(-\epsilon^{-1})$ (bottom panel) respectively.

2.3 Images used in the FFT

Figure S5 shows the background-corrected magneto-optical images used in the calculation of the fast Fourier transform (FFT) presented in Fig. 3(b) in the main text. The blue, orange, green and red points shown in Fig. 3(b) were extracted from the images shown in Fig. S5(a)-(d) respectively.

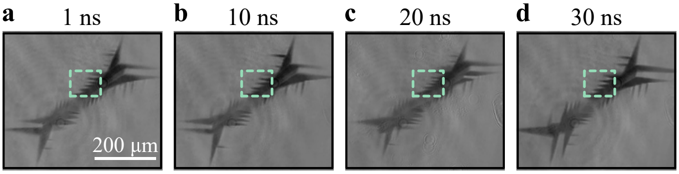

Fig. S5: **(a)-(d)** Background-corrected magneto-optical images used for the FFT calculation, taken at different time delays as indicated. The dashed boxes indicate the region analysed.

2.4 Dynamics of magnetic switching in the rotated sample

Figure S6 shows the temporal evolution of the switched pattern when the thin film of Lu:YIG is rotated by $-30°$ in the plane of the film relative to that used in the main text. By rotating the sample in its plane, the direction of the applied magnetic field is effectively changed. Likewise, the orientation of the probe's polarisation relative to the magnetisation direction of each domain is also altered, modifying the observed magnetic contrast. The magneto-optical images shown in Fig. S6 were obtained with a pump wavelength of 13.0 µm and with an in-plane magnetic field -0.033 mT. Note the appearance of ripples during the first hundreds of nanoseconds after pumping, regardless of the magnetic field's orientation.

References

[1] Malozemoff, A., Slonczewski, J.C.: Magnetic Domain Walls in Bubble Materials: Advances in Materials and Device Research. Academic Press, New York (1979)

[2] Stupakiewicz, A., Davies, C.S., Szerenos, K., Afanasiev, D., Rabinovich, K.S., Boris, A.V., Caviglia, A., Kimel, A.V., Kirilyuk, A.: Ultrafast phononic switching of magnetization. Nat. Phys. **17**(4), 489–492 (2021). https://doi.org/10.1038/s41567-020-01124-9

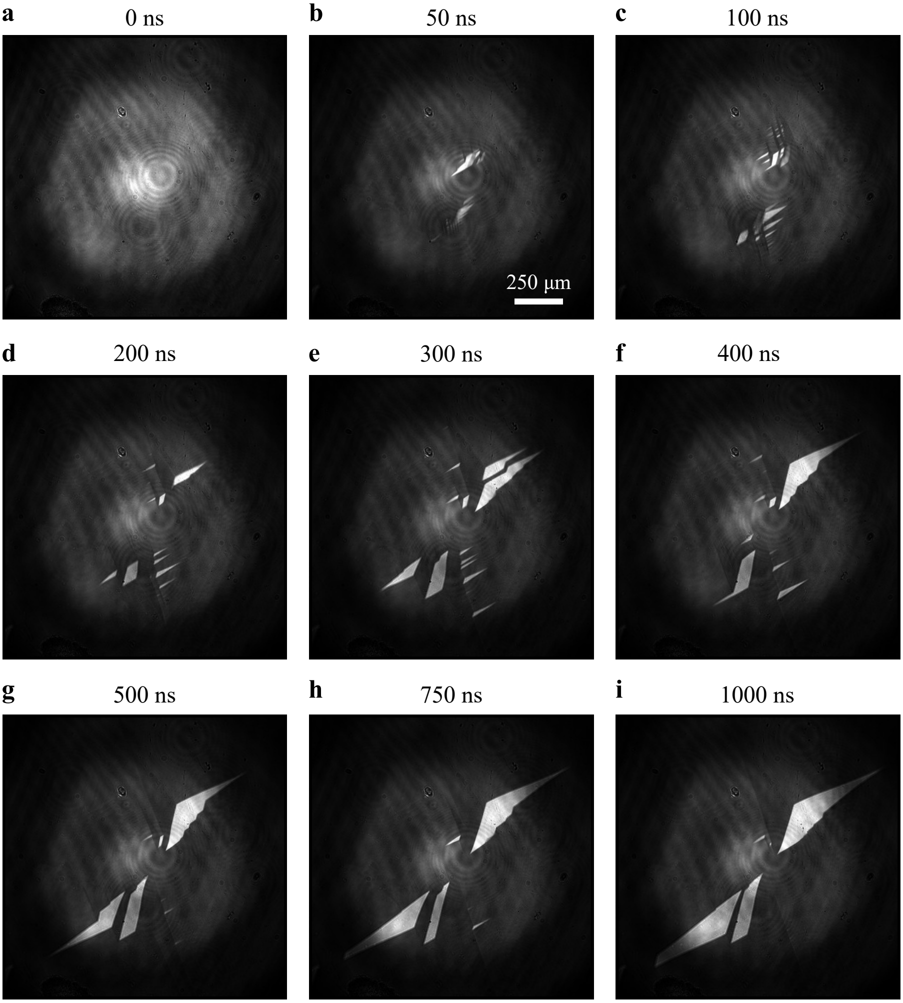

Fig. S6: **(a)-(i)** Magneto-optical images of the switched pattern of magnetisation, taken at the time delays as indicated. These were obtained while applying a static magnetic field of strength 0.033 mT at an angle of $-30°$ relative to that used in Figs. 1-2. The pump wavelength is 13.0 µm. Note that the magnetic contrast here does not distinguish each magnetisation orientation equally well.
